# RETRACTION: Krüppel‐Like Factor 5 Mediates Proinflammatory Cytokine Expression in Lipopolysaccharide‐Induced Acute Lung Injury through Upregulation of Nuclear Factor‐κB Phosphorylation In Vitro and In Vivo

**DOI:** 10.1155/mi/9810135

**Published:** 2026-04-28

**Authors:** Mediators of Inflammation

RETRACTION: H‐L. Chen, I‐W. Chong, Y‐C. Lee, et al., “Krüppel‐Like Factor 5 Mediates Proinflammatory Cytokine Expression in Lipopolysaccharide‐Induced Acute Lung Injury through Upregulation of Nuclear Factor‐κB Phosphorylation In Vitro and In Vivo,” *Mediators of Inflammation*, vol. 2014, (2014). https://doi.org/10.1155/2014/281984.

The above article, published online on 12 August 2014 in Wiley Online Library (wileyonlinelibrary.com), has been retracted by John Wiley & Sons Ltd.

The retraction has been agreed following an investigation by the publisher, which identified a concern related to Figure 6b. More specifically, the bottom right section of the image depicting lung tissue injected with NAC is identical to the top left section of the panel representing the lung tissue subjected to NAC+LPS treatment. As a result, the conclusions of the current article are considered unreliable.

The authors were informed of the decision to retract the article but did not provide a response.

